# Prevalence of Dissociative Convulsions in Patients with Dissociative Disorder in a Tertiary Care Hospital: A Descriptive Cross-sectional Study

**DOI:** 10.31729/jnma.4640

**Published:** 2019-10-31

**Authors:** Jai Bahadur Khattri, Bharat Kumar Goit, Rabindra Kumar Thakur

**Affiliations:** 1 Department of Psychiatry, Manipal College of Medical Sciences, Pokhara, Nepal; 2 Department of Psychiatry, Janaki Medical College, Janakpur, Nepal; 3 Department of Psychiatry, Narayani Hospital, Birgunj, Nepal

**Keywords:** *conversion disorder*, *dissociative disorders*, *Nepal*, *prevalence*, *seizures*

## Abstract

**Introduction:**

Dissociative disorder is one of the common psychiatric problems encountered very frequently in the hospital setting. The aim of the study is to find out the prevalence of dissociative convulsions type in patients suffering from dissociative disorder attending Psychiatry outpatient department of a tertiary care hospital.

**Methods:**

The descriptive cross-sectional study was carried out in a tertiary care hospital from February 2019 to July 2019 after taking ethical approval (MEMG/IRC/210/GA). The patients attending Psychiatry outpatient department of Manipal Teaching Hospital, Pokhara, Nepal were included in the study. The diagnosis of dissociative convulsion and other types of dissociative disorder was done according to the International Classification of Disease–10 Classification of Mental and Behavioural Disorders - Diagnostic Criteria for Research. Statistical Package for Social Sciences version 22 was used for the analysis of the data and point estimate at 95% Confidence interval was calculated along with frequency and proportion for binary data and the analysis was done.

**Results:**

Sixty six patients were included in the study. The prevalence of dissociative convulsion was 86.3% in the present study, at 95% Confidence interval, (78-94.6%). Five patients (7.6%) were found to be suffering from dissociative motor disorders and 4 (6.1%) patients were suffering from trance and possession disorder.

**Conclusions:**

The prevalence of dissociative convulsion type is high in patients suffering from dissociative disorder in the Nepalese context. Future studies should be conducted to understand this disorder and to propose therapeutic guidelines.

## INTRODUCTION

Dissociative disorder is a loss of the normal integration between memories of the past, awareness of identity and immediate sensations, and control of bodily movements.^1^ It is more prevalent in developing countries compared to the developed countries.^2,3^ Estimated prevalence is 5% in general hospital setting and 2.6% in general medicine inpatients.^4,5^ About 2.08% of the inpatients in Nepal were diagnosed as dissociative disorder.^6^

Dissociative convulsions (pseudo seizure) may mimic epileptic seizures very closely.^1^ It is estimated that more than 25 percent of patients receiving a diagnosis of refractory epilepsy do not have epilepsy.^7^ If this condition is not diagnosed early, significant iatrogenic harm may occur.^8^ Although, there are limited studies done at other parts of Nepal, but this type of study has not been done in the Gandaki province of Nepal.

The objective of the study was to study the prevalence of dissociative convulsions type in the patients diagnosed as dissociative disorder.

## METHODS

This descriptive cross-sectional study was conducted in the Psychiatry OPD of Manipal Teaching Hospital, Pokhara. The ethical approval for this study was taken from the ethical review committee of Manipal College of Medical Sciences, Pokhara, Nepal before the start of the study. The study was conducted from February 2019 to July 2019.

n = Z^2^ × p × q/e^2^

   =(1.96)^2^ x (0.78 × 0.22)/(0.1)^2^

   = 0.6589/0.01

   = 65.89

Hence, the sample size is 66.

where,

n = sample size
Z = 1.96 at 95% CI
p = prevalence, 78% (% of dissociative convulsions)
q = 1 – p
d = Margin of error, 10%

Based on the above formula, the minimum sample size was calculated to be 66 and thus the study was conducted in 66 patients. The convenience sampling method was used. Selection and information bias has been minimized as possible. Data entry was done in SPSS, point estimate at 95% CI was calculated along with frequency and proportion for binary data and analysis was done.

The diagnosis of dissociative convulsion and other subtypes of dissociative disorder were done according to the ICD -10 Classification of Mental and Behavioural Disorders- Diagnostic Criteria for Research (ICD-10 DCR).^1^ ICD-10 DCR provides specific criteria for the diagnosis contained in clinical descriptions and diagnostic guidelines, which was produced for general clinical and educational use by psychiatrists and other mental health professionals.^1^

## RESULTS

The prevalence of dissociative convulsion was 86.3% in the present study, at 95% C.I. (78-94.6%).

The diagnosis of respondents as per the type of dissociative disorder according to ICD-10 DCR was done. The totals of 66 patients diagnosed with dissociative disorder were studied. Fifty seven patients (86.3%) were diagnosed as dissociative convulsion disorders. Five patients (7.6%) were found to be suffering from dissociative motor disorders and 4 (6.1%) patients were suffering from trance and possession disorder (Table 1).

**Table 1:** Types of dissociative disorder of the patients. (n=66)

Types of dissociative disorder	n (%)
Trance and possession disorders	4 (6.1)
Dissociative motor disorders	5 (7.6)
Dissociative convulsions	57 (86.3)
TOTAL	66 (100)

## DISCUSSION

Different studies clearly demonstrate that dissociative disorders are a common mental health problem encountered in the clinical practice. Dissociative convulsions markedly impair quality of life of the patients and their close superficial resemblance to epileptic seizures makes them difficult to diagnose. This descriptive cross-sectional study was conducted in Manipal Teaching Hospital, a tertiary care hospital located in Pokhara in the Gandaki province of Nepal. The totals of 66 patients diagnosed as dissociative disorder according to ICD-10 DCR were analysed.

This study showed that the prevalence of dissociative convulsions type of dissociative disorders was 86.3% whereas the prevalence of dissociative motor disorders type and trance and possession disorders type was 7.6% and 6.1% respectively. The study done in Chitwan, Nepal showed that 49% of the patients presented with dissociative convulsions, 15.7% with dissociative motor disorders, 15.7% with dissociative stupor, 11.8% with dissociative anesthesia and sensory loss and 7.8% with trance and possession disorder.^9^ The other study conducted in Dharan, Nepal showed dissociative convulsion in 57% cases, dissociative motor disorder in 9% cases, dissociative stupor in 13% case, and rest cases were diagnosed as mixed dissociative disorder.^10^

In the study conducted in Pakistan, dissociative convulsions (63%) was the most prevalent presentation followed by dissociative motor disorder (24%), mixed dissociative disorder (8%), dissociative anesthesia and sensory symptoms (4%) and trance and possession disorder (1%).^11^

The dissociative convulsion as the most common manifestation of dissociative disorder was also found in other study.^12^ However, two studies reported that dissociative motor disorder was the most common type of presentation among dissociative patients.^13,14^ Mixed dissociative disorder was the most common disorder in one study from Pakistan.^15^ In prevalence study conducted in New York, the most type among dissociative disorder was dissociative amnesia followed by dissociative disorder not otherwise specified, dissociative identity disorder and depersonalization disorder.^16^

The difference in the study findings between different studies could be due to difference in socioeconomic status, culture, lifestyle and level of education of the study populations. In the Nepalese culture, the bodily ill health is more acceptable and respectable than emotional symptoms and only by dramatic appearance of such symptoms can patient gain attention very quickly.

There are limitations of this study. The samples for this study were taken from the teaching hospital of only one metropolitan area. So, further study from different region is suggested.

## CONCLUSIONS

There is high prevalence of dissociative convulsions type among patients suffering from dissociative disorder. This manifestation of the patient suffering from dissociative disorder would help in the proper management of this disabling condition in the long run.

## Conflict of Interest:

None.
